# A randomized, controlled study to assess the efficacy and safety of lotilaner (Credelio™) in controlling ticks in client-owned dogs in Europe

**DOI:** 10.1186/s13071-017-2478-9

**Published:** 2017-11-01

**Authors:** Daniela Cavalleri, Martin Murphy, Wolfgang Seewald, Jason Drake, Steve Nanchen

**Affiliations:** 1 Elanco Animal Health, Mattenstrasse 24a, 4058 Basel, Switzerland; 2 Elanco Animal Health, 2500 Innovation Way, Greenfield, IN 46140 USA

**Keywords:** Credelio, *Dermacentor*, Dog, Efficacy, Europe, Field, *Ixodes*, Lotilaner, *Rhipicephalus*, Ticks

## Abstract

**Background:**

Oral administration of lotilaner flavoured chewable tablets (Credelio™, Elanco) to dogs has been shown to provide a rapid onset of killing activity of infesting ticks, with sustained efficacy for at least 35 days. A study was undertaken in Europe to confirm lotilaner’s safety and anti-tick efficacy in client-owned dogs.

**Methods:**

In this assessor-blinded study, dogs were enrolled at 19 clinics in Germany, Hungary and Portugal. Qualifying households with no more than three dogs were randomized in an approximate 2:1 ratio to a lotilaner or fipronil/(S)-methoprene (FSM) (Frontline® Combo Spot-on, Merial) treatment group. One household dog with at least three live attached ticks was the primary dog. Treatments were dispensed Days 0, 28 (± 2) and 56 (± 2) for owner administration to all household dogs. Tick counts were performed on primary dogs Days 7 (± 1), and ±2 days on Days 14, 21, 28, 42, 56, 70 and 84; supplementary dogs were assessed for safety ± 2 days on Days 28, 56 and 84. Efficacy was assessed by comparing mean Day 0 live attached tick counts with subsequent counts.

**Results:**

The most frequently retrieved ticks were *Ixodes ricinus*, *Dermacentor reticulatus* and *Rhipicephalus sanguineus* (*sensu lato*), with *Ixodes hexagonus* also present. In the lotilaner group (*n* = 127) geometric mean tick count reductions were at least 98% from the first post-treatment visit (Day 7) through Day 56, when efficacy was 100%. For FSM (*n* = 68), efficacy remained at least 96% through Day 84, but at no point were all dogs free of live attached ticks. Mean counts in lotilaner-treated dogs were significantly lower than FSM-treated dogs on Days 7, 42, 70 and 84 (*P* < 0.05). Percent efficacy over all post-enrolment visits was 99.3 and 98.3% for lotilaner and FSM groups, respectively (*t*_(190)_ = 2.23, *P* = 0.0268). Owners successfully administered all treatments, and both products were well-tolerated.

**Conclusion:**

Under European field conditions, lotilaner flavoured chewable tablets administered monthly, were > 98% effective in eliminating live ticks from the first post-treatment assessment (Day 7) through Day 56 and maintained 100% of dogs tick-free on Days 70 and 84. Lotilaner was safe, providing superior tick control to FSM administered according to the same schedule.

**Electronic supplementary material:**

The online version of this article (10.1186/s13071-017-2478-9) contains supplementary material, which is available to authorized users.

## Background

The broadening geographical spread and increasing abundance in Europe of the ixodid ticks, *Ixodes ricinus*, *Dermacentor reticulatus* and *Rhipicephalus sanguineus* (*sensu lato*), has been linked to changes in climate that have provided favourable epidemiological conditions, and to changing human behaviours that increase the risk of tick exposure of humans and pets [1–5]. As a result, ticks are increasingly a threat to human and animal health, as a result of a direct pathogenic effect and most importantly through the disease-producing organisms they transmit, including those responsible for tick-borne encephalitis, babesiosis, anaplasmosis and ehrlichiosis [5–7]. To reduce the risk of the establishment of canine tick infestations, it is therefore important that veterinarians and owners have effective options that can provide reliable and sustained effectiveness throughout the recommended between-treatment interval.

A new option for controlling ticks and fleas is the isoxazoline compound lotilaner. The isoxazolines act systemically to kill the fleas and ticks that infest dogs by targeting distinct binding sites on insect and acarine γ-aminobutyric acid (GABA)- and glutamate-gated chloride-ion channels to cause parasite paralysis and death [8, 9]. Lotilaner has been shown to bind selectively to these sites in invertebrates, but not in dogs [9]. Other members of the family of isoxazoline parasiticides are afoxolaner and fluralaner which first received regulatory approval for use in dogs in 2014, and sarolaner which was approved for use in dogs in 2015. As the most recent member of the family to be approved, lotilaner is a rapid-onset ectoparasiticide that is presented in a flavoured chewable tablet formulation (Credelio™, Elanco) for dogs. Laboratory studies demonstrated that lotilaner has been shown to quickly begin killing induced infestations with *I. ricinus*, and to sustain activity against new infestations with *Ixodes scapularis*, *I. ricinus*, *Dermacentor variabilis*, *D. reticulatus* and *R. sanguineus* (*s.l.*) for at least 35 days, and *Amblyomma americanum* for at least 28 days [10–12]. Similar rapid onset, sustained activity has also been demonstrated against flea infestations [13, 14].

To confirm that these promising results were relevant to natural tick infestations, a study was designed to evaluate the safety and efficacy of lotilaner flavoured chewable tablets against ticks on client-owned dogs in Europe. In three countries, i.e. Germany, Hungary and Portugal, lotilaner was administered orally, by owners, once every 4 weeks for a total of three treatments within the dose range of 20.2 to 40.7 mg/kg body weight to dogs naturally infested with ticks. A topical formulation of fipronil/(S)-methoprene (FSM) (Frontline® Combo, Merial) was used as a positive-control comparator. Assessments were made of the efficacy and safety of each product.

## Methods

This assessor-blinded, positive controlled, randomised, multicentre, non-inferiority clinical field study was conducted in compliance with local and national regulatory requirements in Germany, Hungary and Portugal; in compliance with the VICH guideline on Good Clinical Practice (GCP; VICH GL 9) and Directive 2001/82/EC as amended; The Rules Governing Medicinal Products in the European Union, Volume VIIA: Guidelines for the testing of veterinary medicinal products: Demonstration of Efficacy of Ectoparasiticides, 7AE17a, page 215–222; EMEA/CVMP/EWP/ 005/2000-Rev.2: Guideline for the testing and evaluation of the efficacy of antiparasitic substances for the treatment and prevention of tick and flea infestation in dogs and cats, 12 Nov 2007; and consistent with the guidelines of the World Association for the Advancement of Veterinary Parasitology [15, 16].

### Animals

Dogs were selected for the study on Day 0 after being diagnosed with infestations of at least three live attached ticks. Tick counts were performed on a single dog from each household (the primary dog). Only households with dogs satisfying all of the inclusion criteria and for which none of the exclusion criteria applied were selected for inclusion. Supplementary dogs in any enrolled household were included regardless of tick infestation. To qualify for inclusion, a household could have no more than three dogs, all of which were required to be healthy, or with conditions judged not to interfere with the study objectives, to be at least 8 weeks old, and to weigh at least 2 kg.

A household was excluded from the study if it contained dogs that were pregnant or lactating, or that were intended for breeding within 4 months following the last treatment administration. Households would be removed from the study at any time at the discretion of the investigator or study sponsor for reasons that included protocol non-compliance (for instance, treatment with a study-proscribed product such as one that had any efficacy against ticks), the appearance of concomitant disease, or development of a serious adverse event that was incompatible with continuation in the study. Supplementary dogs in each household were treated with the same product as the primary dogs.

All dogs were kept with their owners under their usual housing conditions before, during and after the study. Because FSM was applied topically, bathing/immersion in water within 2 days after application and more frequent bathing than once a week was to be avoided. Dogs were not allowed to swim in watercourses for 2 days after application, and more frequent bathing than once a week was to be avoided, wherever possible and any water contact was to be documented by the owner.

### Randomisation and treatment

Within each clinic, dogs were randomised by household to the treatment groups in the sequence of inclusion designated by the randomization plan, using a block design and a 2:1 ratio (lotilaner:FSM), with a total enrolment target of 180 primary dogs. The first household dog presenting with an infestation of at least three ticks was the primary dog on which all tick counts would be used for efficacy calculations. All dogs, including supplementary household dogs, were observed for safety assessment.

All dogs from any household were randomised to the same treatment group:

- Group 1 households were dispensed lotilaner flavoured chewable tablets (Credelio, Elanco), available in five tablet sizes (56.25 mg, 112.5 mg, 225 mg, 450 mg and 900 mg lotilaner), to be administered on the basis of each household dog’s body weight to achieve a dose rate between a minimum of 20.2 to a maximum of 40.7 mg/kg, in compliance with the dosing table for the commercial product. At the initial visit and at the second and third visits, the dispenser in each clinic provided the appropriate number of tablets for each household dog to be treated on a single occasion on each of Days 0, 28 (± 2), and 56 (± 2). Owners were instructed to feed their dogs within 30 min prior to treatment;
- Group 2 households were dispensed a formulation of fipronil 10%/(S)-methoprene 0.9% (Frontline Combo, Merial), available in four sizes (0.67 ml, 1.34 ml, 2.68 ml or 4.02 ml), for at-home application on each of Days 0, 28 (± 2), and 56 (± 2). Owners were instructed to apply the product according to label.

### Study assessments

Physical examinations and body weight measurements were completed on each primary dog at each visit, and on supplementary dogs on Days 0, and ± 2 days on each of Days 28, 56 and 84. Blood and urine samples were collected from all dogs for clinical pathology evaluations on Days 0 and 84 (or earlier for dogs prematurely exiting the study).

For primary dogs, tick counts and removal by the blinded clinic staff, trained in study protocol procedures, were completed on Day 7 (± 1), and ± 2 days on each of Days 14, 21, 28, 42, 56, 70 and 84. Attached live and dead ticks were placed in separate vials for later differentiation. The efficacy of each product was evaluated based on live, attached tick counts at each time point. To generate an indication of environmental tick pressure during the study, the numbers of dogs and cats diagnosed as treated for tick infestations at study clinics was recorded.

The three study populations for assessments were: the safety population, consisting of all dogs, primary and supplementary, that were randomised to a treatment group and that received at least one dose of either study product; the intent-to-treat (ITT) population consisting of all primary dogs in each treatment group; and the per-protocol (PP) population, consisting of all primary dogs without major protocol violations. The analyses of efficacy were conducted on both ITT and PP populations. For sex, age, body weight, breed, husbandry, animal spends time indoor/outdoor, country, summary statistics and/or frequencies were calculated and the two groups were compared with a non-parametric test (Kruskal-Wallis, Mann-Whitney, or Fisher’s exact test, depending on the parameter). Safety was assessed according to any observations by an owner or study staff of adverse events, to changes in body weight, and to laboratory results of urinalysis, haematology and serum chemistry.

The efficacy of each treatment was assessed by comparing mean baseline tick counts on Day 0 with those from visits after the first treatment administration and by comparing the overall reduction in mean tick counts over the entire treatment period. Efficacy was determined on the basis of the percent reduction in counts from pre- to post-dosing within each treatment group. Percent efficacy at each counting time point after dosing was calculated as follows:

$$ \mathrm{Percent}\  \mathrm{efficacy}=100\ \left(\mathrm{M}0-\mathrm{MD}\right)/\left(\mathrm{M}0\right) $$

where M0 is the mean tick count on Day 0 and MD is the mean tick count on actual day; tick counts are based on live attached ticks.

Calculations were performed using geometric and arithmetic means. Calculation of geometric means involved taking the logarithm of the tick count of each dog. If any of the counts were equal to zero, a one was added to the count for every animal in the group and then subtracted from the resultant mean prior to calculating percent efficacy. Statistical analysis was completed for: tick counts for live attached ticks; reduction of tick counts versus baseline for live attached ticks; cure rates (percentage of dogs with zero tick counts) for live attached ticks.

Treatment groups were compared by analysis of (co)variance (AN(C)OVA) methods if the assumption of normal distribution was satisfied on original scale or after possible log-transformation. In the ANCOVA, the number of dogs per household was used as a covariate. Non-inferiority was claimed if the lower limit of the two-sided 95% confidence interval (CI) for the ratio of tick counts for lotilaner, divided by the same value for FSM, provided 97.5% confidence that tick counts from lotilaner treatment were not higher than those from FSM treatment, up to a non-inferiority margin of 15%. Superiority was claimed if the 95% CI lay completely within the interval (0, 1), providing 97.5% confidence that tick counts from lotilaner treatment were lower than those from FSM treatment. If the assumption of normal distribution was not satisfied, Mann-Whitney U tests were performed. The numbers of AEs by clinical sign were compared between treatment groups using Fisher’s exact test. All calculations were carried out using the software SAS®, Version 9.2.2.

### Translations

Spanish translation of the article is available in Additional file 1. French translation of the Abstract is available in Additional file 2.

## Results

### Dogs and efficacy against ticks

One hundred and ninety five primary dogs (households) were enrolled into the study, 127 in the lotilaner group and 68 in the FSM group, at seven clinics in Germany, six clinics in Hungary, and six clinics in Portugal. The first enrolment was on April 14, 2014, and the final follow up was on August 21, 2014. Owners reported administering treatments per schedule, and all treatments were successfully administered by owners. Across the safety population the administered lotilaner dose rate ranged from 20.2 to 40.7 mg/kg. There were no owner reports that resulted in exclusion of a dog because of a protocol violation due to water exposure or bathing. None of the dogs was completely excluded from the statistical analysis: three primary dogs in each group were partially excluded from the PP analysis because of a delayed Day 84 visit; eleven dogs (six primary, five supplementary) did not complete the study on the schedule completion day (day 84 ± 2). Of the non-completing primary dogs, one dog in the lotilaner group and two FSM-group dogs were withdrawn because of owner non-compliance with the protocol; and two and one dogs in the lotilaner and FSM groups, respectively, were withdrawn because of serious adverse events (AEs), detailed in the Safety section.

**Fig. 1:**
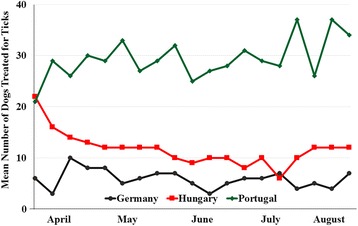
Mean number of non-study dogs treated for tick infestations; average over study sites within countries at weekly intervals throughout the study

Groups were homogeneous for age, weight and sex distribution, and there were no statistically significant baseline differences between treatment groups in any demographic variable (Table 1). Forty-eight different breeds were included in the study, of which the most frequently enrolled were Labrador retriever (*n* = 12), German shepherd (*n* = 9), and Golden retriever (*n* = 8). Tick species infesting dogs in each group were balanced, with no significant difference between groups. Throughout the study, in each country the number of non-study dogs that were treated for tick infestations at enrolling clinics verified the presence of a tick challenge throughout the study period (Fig. 1). Similar observations at study clinics of treatments of cats for tick infestations were made in each country throughout the study period.

**Table 1:** Demographics of enrolled dogs (efficacy population)

		Lotilaner (*n* = 127)	Fipronil/(S)-methoprene (*n* = 68)
Age (years)	Mean ± SD	4.6 ± 3.5	5.1 ± 4.0
	Range	0.2−14.0	0.3−16.0
Weight (kg)	Mean ± SD	22.1 ± 13.8	20.4 ± 13.6
	Range	2.4−62.0	2.2−73.9
Sex	Female	47 (37.0%)	27 (39.7%)
	Male	80 (63.0%)	41 (60.3%)
Location	Countryside	83 (65.4%)	48 (70.6%)
	Urban	44 (34.6%)	20 (29.4%)
Primary dog spends time	Mostly indoors	41 (32.3%)	26 (38.2%)
	Mostly outdoors	86 (67.7%)	42 (61.8%)

*Abbreviation*: *SD* standard deviation

In study dogs, the most commonly identified tick species were *I. ricinus*, *R. sanguineus* (*s.l.*) and *D. reticulatus*, with *Ixodes hexagonus* also found on 12 study dogs (Table 2). In Germany, the most commonly isolated species was *I. ricinus*, with occasional identification of *I. hexagonus* and *D. reticulatus.* There was identification of just one *R. sanguineus* (*s.l.*) from one dog in Germany. In Hungary, both *I. ricinus* and *D reticulatus* were commonly identified, *R. sanguineus* (*s.l.*) ticks were collected from three dogs and there was a single identification of *Haemaphysalis concinna*. In Portugal where tick counts on each dog were much greater than in the other countries, the dominant tick species was *R. sanguineus* (*s.l.*). In this country, other species were found on only two dogs, one with one male and one female *D. reticulatus*, and one with 20 *I. hexagonus* females. Despite treatment with FSM, this dog remained infested with *I. hexagonus* at different points through Day 84, when two live nymphs were recovered. All ten lotilaner-treated study dogs presenting on Day 0 with at least one *I. hexagonus* (all in Germany) were free of this species at all subsequent assessments.

**Table 2:** Percent of study dogs infested with different tick species at baseline

	Lotilaner (*n* = 127)		Fipronil/(S)-methoprene (*n* = 68)	
	% infested	Total live ticks	% infested	Total live ticks
*Ixodes ricinus*	64.6	294	60.3	136
*Rhipicephalus sanguineus*	36.2	1147	33.8	216
*Dermacentor reticulatus*	27.6	85	32.4	58
*Ixodes hexagonus*	7.9	14	2.9	23
*Ixodes* spp.	0.8	1	1.4	1
*Haemaphysalis concinna*	0.8	1	0	0

**Fig. 2:**
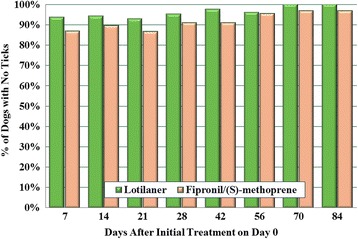
Percent of dogs that were free of live ticks after treatment with either lotilaner or fipronil/(S)-methoprene on Days 0, 30 and 60

There was no statistically significant difference between the ITT and PP population, and only the PP population efficacy results are presented here. Baseline geometric mean tick counts in the lotilaner and fipronil groups were 6.06 and 5.01, respectively (Table 3). At the first post-treatment assessment (Day 7) the geometric mean tick count in lotilaner-treated dogs (0.07) was significantly lower (*t*_(190)_ = 2.37, *P* = 0.0190) than that of the FSM-treated dogs (0.17), and remained lower on all but one of the counts through the final assessment on Day 84. Other points at which mean lotilaner-group counts were significantly lower than FSM-group counts were Days 42 (*t*_(186)_ = 2.46, *P* = 0.0148), 70 (*t*_(184)_ = 2.04, *P* = 0.0425), and 84 (*t*_(178)_ = 2.33, *P* = 0.0209). In the lotilaner group, efficacy based on geometric means remained greater than 98% (based on arithmetic means at least 95%) throughout the study, while in the FSM group efficacy remained at greater than 96% (for arithmetic means greater than 93%) (Table 4). More than 95% of lotilaner-treated dogs were free of live attached ticks from the first assessment (Day 7) to Day 63, and on Days 70 and 84 all dogs in this group were completely free of live ticks (Fig. 2). Investigation of efficacy against the different infesting tick species in study dogs did not reveal any clinically relevant departures from the overall tick efficacy results for either product.

**Table 3:** Mean live attached tick counts on each treatment group (per protocol population)

	Day of study								
	0	7	14	21	28	42	56	70	84
Lotilaner (*n* = 127)									
Geometric mean	6.06	0.07^a^	0.07	0.10	0.06	0.02^b^	0.05	0.00^c^	0.00^d^
Arithmetic mean (SD)	11.61 (28.76)	0.15 (0.94)	0.15 (0.76)	0.58 (5.27)	0.15 (0.82)	0.04 (0.30)	0.28 (2.78)	0.00 (0.00)	0.00 (0.00)
Fipronil/(S)-methoprene (*n* = 68)									
Geometric mean	5.01	0.17^a^	0.11	0.13	0.09	0.11^b^	0.04	0.02^c^	0.03^d^
Arithmetic mean (SD)	6.31 (8.06)	0.40 (1.47)	0.19 (0.63)	0.22 (0.65)	0.18 (0.89)	0.20 (0.68)	0.06 (0.30)	0.03 (0.17)	0.05 (0.28)

*Abbreviation*: *SD* standard deviation

Numbers within columns with the same superscript are significantly different: ^a^ *t*_(190)_ = 2.37, *P* = 0.0190; ^b^ *t*_(186)_ = 2.46, *P* = 0.0148; ^c^ *t*_(184)_ = 2.04, *P* = 0.0425; ^d^ *t*_(178)_ = 2.33, *P* = 0.0209

**Table 4:** Percent effectiveness of lotilaner and fipronil/(S)-methoprene (per protocol population)

	Day of study							
	7	14	21	28	42	56	70	84
Lotilaner (*n* = 127)								
Geometric mean	98.9^a^	98.9	98.4	98.9	99.6^b^	99.2	100.0	100.0
Arithmetic mean	98.7	98.7	95.0	98.7	99.7	97.6	100.0	100.0
Fipronil/(S)-methoprene (*n* = 68)								
Geometric mean	96.6^a^	97.8	97.3	98.3	97.9^b^	99.2	99.6	99.4
Arithmetic mean	93.7	96.9	96.5	97.2	96.9	99.0	99.5	99.2

Numbers within columns with the same superscript are significantly different:^a^ *t*_(190)_ = 3.14, *P* = 0.0020; ^b^ *t*_(186)_ = 2.39, *P* = 0.0180

In the lotilaner group, the average percent reduction in live attached tick counts over all post-enrolment visits was 99.3% (geometric mean) and 98.6% (arithmetic mean), and for the FSM group 98.3 and 97.4%. It was shown (with a 97.5% confidence limit) that tick counts in the lotilaner group were not higher than tick counts in the FSM group, up to a non-inferiority margin of 15%. In addition to demonstrating non-inferiority, these results also show superiority of lotilaner over FSM (*t*_(190)_ = 2.23, *P* = 0.0268).

Two dogs that were randomized to the lotilaner group had pre-treatment burdens of more than 200 *R. sanguineus* (*s.l.*). One of these dogs was free of live attached ticks at the first post-treatment visit (Day 7) and remained so thereafter, and the other remained free of live attached ticks following administration of the second treatment. In the FSM group, the highest baseline tick count was 64 (*R. sanguineus* (*s.l.*), and occasional findings on this dog of low numbers of this species continued throughout the study.

### Safety

Including supplementary dogs in each household, the safety population comprised 192 dogs treated with lotilaner and 94 with FSM. Both study treatments were well tolerated. The most commonly reported AEs were digestive tract disorders (diarrhea, emesis, gastric dilation) which occurred in 3.6% and 1.1% of lotilaner- and FSM-group dogs respectively (Table 5). There were no between-group statistical differences in any of these signs. One dog in the lotilaner group, a 14-year-old female long haired Dachshund had a history at enrolment of a suspected (chronic) nephritis based on the Day 0 testing (azotaemia). On Day 20, the dog presented with polydipsia and polyuria but received no concomitant treatment except a change to a specialized kidney diet. From Day 28 no polydipsia/polyuria was observed, but additional bloodwork revealed an increase in serum creatinine. This was recorded as another AE (abnormal test result - azotaemia) on Day 30 due to worsening compared to Day 0. At study end, the azotaemia had neither worsened significantly nor resolved, while serum phosphate and potassium levels had increased significantly, leading to documentation of two further AEs. There were no statistically significant differences between the two treatment groups for any of the reported clinical signs of AEs or serious AEs.

**Table 5:** Adverse events observed in at least 1% of dogs in either group

	Lotilaner (*n* = 192)	Fipronil/(S)-methoprene (*n* = 94)	Comparison (Fischer’s exact test)
Emesis	3.1%	0.0%	*z* = 1.73, *P* = 0.1825
Diarrhea	1.0%	0.0%	*z* = 0.99, *P* = 0.5572
Gastric dilation	0.0%	1.1%	*z* = 1.42, *P* = 0.3287
Abnormal test result	1.6%	2.1%	*z* = 0.34, *P* = 1.0000

There were four serious AEs observed in study dogs, none of which were attributed to treatment. In the lotilaner group, two primary dogs were affected, one of which presented with a cauda equina syndrome on Day 5 and was euthanised at the owner’s request; the other died after being involved in a car accident. A supplementary lotilaner-group dog with a pre-study dilative cardiomyopathy was found dead by the owner on Day 75. In the FSM group, a primary dog remained in the study after recovering with sequelae from surgery to correct a hind limb paresis due to a disc prolapse with onset on Day 27.

No significant difference was seen between the treatment groups for haematology or urinalysis results. Serum chemistry results showed a significant difference for cholesterol on day 84, with lower values in the FSM group (*t*_(163)_ = 2.81, *P* = 0.0056), but the mean value remained within the normal reference range and was not associated with any clinical signs of illness. All other measured parameters were not significantly different.

The only significant body weight difference between the two treatment groups was on Day 7 (*t*_(189)_ = 2.19, *P* = 0.0301) when the lotilaner group had a higher mean weight than the FSM group (corrected for baseline body weight). The mean body weight of each treatment group did not change notably during the study period but increased slightly from day 0 to day 84 in both groups.

Multivalent and monovalent rabies vaccinations and concomitant medications were administered to lotilaner-group dogs with no associated adverse events. Reported concomitant systemic medications were amoxicillin, benzylprocaine penicillin/dihydrostreptomycin, benazepril hydrochloride, domperidone, furosemide, imepitoin, meloxicam, milbemycin oxime, pimobendan, praziquantel, praziquantel/pyrantel pamoate/febantel, robenacoxib, and spironolactone.

## Discussion

The frequency of tick infestations in this study substantiates the ongoing risk of tick challenge throughout a range of European climates, and emphasizes the need for effective tick treatment and prevention measures. That there was a sustained tick challenge to enrolled dogs is supported by the presentation of non-study tick-infested dogs to participating clinics throughout the study, and to the persistence of tick numbers on treated dogs. Additionally, most dogs were classified as outdoor dogs from rural environments, situations in which dogs are likely to be exposed to tick challenge.

The dominant species that were identified in this study, i.e. *I. ricinus*, *R. sanguineus* (*s.l.*) and *D. reticulatus*, are consistent with other reports, although there is no support for the suggestion of the northward migration of *R. sanguineus* (*s.l.*) beyond Mediterranean areas [17–19]. There were well defined regional differences in the challenge, with *Ixodes*, predominantly *I. ricinus* but also *I. hexagonus*, the most commonly observed tick species in Germany, a balance of *I. ricinus* and *D. reticulatus* in Hungary, and *R. sanguineus* was dominant in Portugal. The total tick counts on dogs in Portugal due to *R. sanguineus* (*s.l.*) infestations were far greater in number than those that occurred with other species in Germany and Hungary.

The fact that all dogs were successfully treated by their owners, with no compliance concerns indicates that the lotilaner flavoured chewable tablet formulation is readily accepted by dogs. This conclusion is supported by another European field study in which lotilaner was found to be palatable for dogs, and a field study in the United States when there was no practical difference shown between lotilaner and afoxalaner in voluntary acceptance of owner-offered treatments [20, 21].

Regardless of the country, region or infesting tick species or size of tick burden, lotilaner provided high and sustained efficacy throughout the 84-day study. At study-end all lotilaner-treated dogs were completely free of live ticks, and geometric mean tick counts were significantly lower than those of the FSM group overall and on Days 7, 42 and 70. Lotilaner’s sustained high efficacy against natural tick infestations confirms studies with induced infestations in which efficacy against the three dominant European tick species was shown to be at least 95% through 35 days after treatment [11]. Lotilaner was well tolerated across breeds and environments, and was safely administered with a range of common medications and vaccines. These results are consistent with a parallel field study in Europe assessing lotilaner efficacy against fleas [20]. In that study 97.5% of lotilaner-treated dogs were cleared of live fleas within 4 weeks following the third of three monthly treatments, and lotilaner was significantly more effective than fipronil.

In this study, the arithmetic mean tick count reductions in the FSM group ranged from 93.7% on Day 7 to 99.5% on Day 84. These results are similar to those reported in two similar field studies in Europe comparing fipronil to other isoxazolines. In a comparison with sarolaner, the arithmetic mean percent reductions in the fipronil group ranged from 88.5 to 98.1%, with sarolaner showing significantly greater reductions in tick counts on two occasions (Days 30 and 60) [17]. In a comparative study with fluralaner, fipronil anti-tick efficacy based on geometric means ranged from 97.6 to 100%, with no reported significant differences between groups [18]. The cumulative results of these studies therefore suggest that while the tick-killing activity of fipronil remains high, the isoxazolines have the potential to provide a superior level of tick control, which may be of relevance in the prevention of tick-borne pathogen transmission and in owner perception of product effectiveness.

## Conclusion

The results of this study, undertaken in a diverse range of client-owned dogs and geographical regions, demonstrate that under a wide range of field conditions in Europe, a single lotilaner treatment resulted in a 98.9% reduction in live attached tick counts by the time of the first assessment at 7 days after the first treatment. This high level of efficacy was sustained following three monthly treatments. At the end of the study, 28 days following the final treatment, all lotilaner-treated dogs were free of live ticks. The results demonstrate that lotilaner has consistent and sustained high efficacy against ticks that infest dogs under European conditions. The results also demonstrate that lotilaner, is easy for owners to administer, and is well tolerated and safe for use with a range of vaccinations and concomitant medications. The tick control provided by lotilaner was superior to that provided by a topically applied formulation of fipronil/(S)-methoprene.

## Additional files

Additional file 1: Spanish translation of the article. (PDF 154 kb)

Additional file 2: French translation of the Abstract. (PDF 37 kb)

## Abbreviations

AE: Adverse event
AN(C)OVA: Analysis of (co)variance
CI: Confidence interval
EMEA/CVMP: EMEA/CVMP/EWP European Medicines Agency/ Committee for Medicinal Products for Veterinary Use
GABA: γ-aminobutyric acid
ITT: Intent-to-treat
PP: Per protocol
SD: Standard deviation
VICH: International Cooperation on Harmonization of Technical Requirements for Registration of Veterinary Medicinal Products

## References

[1] Jaenson TG, Jaenson DG, Eisen L, Petersson E, Lindgren E (2012). Changes in the geographical distribution and abundance of the tick *Ixodes ricinus* during the past 30 years in Sweden. Parasit Vectors.

[2] Zając Z, Katarzyna B, Buczek A (2016). Factors influencing the distribution and activity of *Dermacentor reticulatus* (F.) ticks in an anthropopressure-unaffected area in central-eastern Poland. Ann Agric Environ Med.

[3] Abdullah S, Helps C, Tasker S, Newbury H, wall R (2016). Ticks infesting domestic dogs in the UK: a large-scale surveillance programme. Parasit Vectors.

[4] Földvári G, Široký P, Szekeres S, Majoros G, Sprong H (2016). *Dermacentor reticulatus*: a vector on the rise. Parasit Vectors.

[5] Randolph SE (2004). Evidence that climate change has caused ‘emergence’ of tick-borne diseases in Europe?. Int J Med Microbiol.

[6] Kilpatrick AM, Randolph SE (2012). Drivers, dynamics, and control of emerging vector-borne zoonotic diseases. Lancet.

[7] Jaenson TG, Hjertqvist M, Bergström T, Lundkvist A (2012). Why is tick-borne encephalitis increasing? A review of the key factors causing the increasing incidence of human TBE in Sweden. Parasit Vectors.

[8] Ozoe Y, Asahi M, Ozoe F, Nakahira K, Mita T (2010). The antiparasitic isoxazoline A1443 is a potent blocker of insect ligand-gated chloride channels. Biochem Biophys Res Commun.

[9] Rufener L, Danelli V, Bertrand D, Sager H. The novel isoxazoline ectoparasiticide lotilaner (Credelio^TM^): a non-competitive antagonist specific to invertebrates γ-aminobutyric acid-gated chloride channels (GABACls). Parasit Vectors (In press). 10.1186/s13071-017-2470-4 PMC5664438 29089046

[10] Murphy M, Cavalleri D, Seewald W, Drake J, Nanchen S. Laboratory evaluation of the speed of kill of lotilaner (Credelio^TM^) against *Ixodes ricinus* ticks on dogs. Parasit Vectors. 2017. (In press). 10.1186/s13071-017-2467-z PMC5664588 29089039

[11] Cavalleri D, Murphy M, Gorbea RL, Seewald W, Drake J, Nanchen S. Laboratory evaluations of the immediate and sustained effectiveness of lotilaner (Credelio^TM^) against three common species of ticks affecting dogs in Europe. Parasit Vectors. 2017. (In press). 10.1186/s13071-017-2477-x PMC5664927 29089050

[12] Murphy M, Garcia R, Karadzovska D, Cavalleri D, Snyder D, Seewald W, et al. Laboratory evaluations of the immediate and sustained effectiveness of lotilaner (Credelio™) against four common species of ticks affecting dogs in North America. Parasit Vectors. 2017. (In press). 10.1186/s13071-017-2476-y PMC5664823 29089057

[13] Cavalleri D, Murphy M, Seewald W, Drake J, Nanchen S. Assessment of the onset of lotilaner (Credelio^TM^) speed of kill of fleas on dogs. Parasit Vectors. 2017. (In press). 10.1186/s13071-017-2474-0 PMC5664436 29089066

[14] Cavalleri D, Murphy M, Seewald W, Drake J, Nanchen S. Assessment of the speed of flea kill of lotilaner (Credelio^TM^) throughout the month following oral administration to dogs. Parasit Vectors. 2017. (In press). 10.1186/s13071-017-2466-0 PMC5664906 29089019

[15] European Medicines Agency, Committee for Medicinal Products for Veterinary Use, 2000. Guideline on Good Clinical Practices. VICH Topic GL9 http://www.ema.europa.eu/docs/en_GB/document_library/Scientific_guideline/2009/10/WC500004343.pdf. Accessed 12 Jan 2017.

[16] Marchiondo AA, Holdsworth PA, Fourie LJ, Rugg D, Hellmann K, Snyder DE, et al. World Association for the Advancement of veterinary Parasitology (W.A.A.V.P.) 2nd. Ed.: Guidelines for evaluating the efficacy of parasiticides for the treatment, prevention and control of flea and tick infestations on dogs and cats. Vet Parasitol. 2013;194:84–97. 10.1016/j.vetpar.2013.02.003 23741753

[17] Becskei C, De Bock F, Illambas J, Mahabir SP, Farkas R, Six RH (2016). Efficacy and safety of a novel oral isoxazoline, sarolaner (Simparica™) in the treatment of naturally occurring flea and tick infestations in dogs presented as veterinary patients in Europe. Vet Parasitol.

[18] Rohdich N, Roepke RK, Zschiesche E (2014). A randomized, blinded, controlled and multi-centered field study comparing the efficacy and safety of Bravecto (fluralaner) against Frontline (fipronil) in flea- and tick-infested dogs. Parasit Vectors.

[19] Beugnet F, Marié JL (2009). Emerging arthropod-borne diseases of companion animals in Europe. Vet Parasitol.

[20] Cavalleri D, Murphy M, Seewald W, Drake J, Nanchen S. A randomized, controlled field study to assess the efficacy and safety of lotilaner tablets (Credelio^TM^) in controlling fleas in client-owned dogs in European countries. Parasit Vectors. 2017. (In press). 10.1186/s13071-017-2479-8 PMC5664837 29089065

[21] Karadzovska D, Chappell K, Coble C, Murphy M, Cavalleri D, Wiseman S, Drake J, Nanchen S. A randomized, controlled field study to assess the efficacy and safety of lotilaner flavored chewable tablets (Credelio^TM^) in eliminating fleas in client-owned dogs in the USA. Parasit Vectors. 2017. (In press). 10.1186/s13071-017-2469-x PMC5664423 29089063

