# Supplementary material for: A randomized, controlled study to assess the efficacy and safety of lotilaner (Credelio™) in controlling ticks in client-owned dogs in Europe
Source: Parasit Vectors. 2017 Nov 1;10:531. doi: 10.1186/s13071-017-2478-9 (PMC5664821; doi:10.1186/s13071-017-2478-9)
Supplement: Supplementary file 1 — Spanish translation of the article. (PDF 154 kb) [file 13071_2017_2478_MOESM1_ESM.pdf]

Un estudio controlado y aleatorizado para evaluar la eficacia y seguridad de lotilaner (Credelio™) en el control de garrapatas en perros con dueño en Europa

Daniela Cavalleri[1], Martin Murphy[1], Wolfgang Seewald[1], Jason Drake[2]* y Steve Nanchen[1]

[1]Elanco Animal Health, Mattenstrasse 24a, CH-4058 Basilea, WRO-1032.2.58, Suiza WRO.
[2]Elanco Animal Health, 2500 Innovation Way, Greenfield, IN 46140, EUA

*****Correspondencia: drake_jon_j@elanco.com

E-mails:
Daniela Cavalleri[1] e-mail: cavalleri_daniela_a@elanco.com
Martin Murphy[1] e-mail: murphy_martin_gerard@elanco.com
Wolfgang Seewald[1] e-mail: seewald_wolfgang@elanco.com
Jason Drake[2] e-mail: drake_jon_j@elanco.com
Steve Nanchen[1] e-mail: nanchen_steve@elanco.com

Resumen

Antecedentes: Se ha demostrado que administración oral de lotilaner como tabletas masticables saborizadas (Credelio™, Elanco) a perros provee un rápido inicio de la actividad para matar garrapatas infestantes, con una eficacia sostenida de al menos 35 días. Un estudio fue llevado a cabo en Europa para confirmar la seguridad de lotilaner y la eficacia anti-garrapata en perros con dueño.

Métodos: En un estudio ciego para el asesor, se enrolaron perros de 19 clínicas en Alemania, Hungría y Portugal. Los hogares que calificaron no tenían más de tres perros que se aleatorizaron en una proporción aproximada de 2:1 a un grupo de tratamiento con lotilaner o con fipronil fipronil/(S)-metopreno (FSM) (Frontline® Combo Spot-on, Merial). Un perro de casa con al menos tres garrapatas adheridas vivas fue el perro primario. Los tratamientos se dispensaron los Días 0, 28 (± 2) y 56 (± 2) para que el dueño realizara la administración a todos los perros de su hogar. Los conteos de garrapatas se realizaron en los perros primarios los Días 7 (± 1), y ± 2 días en los Días 14, 21, 28, 42, 56, 70 y 84; los perros suplementarios fueron evaluados para seguridad ± 2 días en los Días 28, 56 y 84. La eficacia

se evaluó comparando la media de los conteos de garrapatas adheridas en el Día 0 con conteos subsecuentes.

Resultados: Las garrapatas más frecuentemente recuperadas fueron *Ixodes ricinus*, *Dermacentor reticulatus* y *Rhipicephalus sanguineus* (*sensu lato*), con *Ixodes hexagonus* también presente. En el grupo de lotilaner (n = 127) la media geométrica de las reducciones en el conteo de garrapatas fue de al menos el 98% de la primera visita post-tratamiento (Día 7) hasta el Día 56, cuando la eficacia fue del 100%. Para FSM (n = 68), la eficacia permaneció en al menos 96% hasta el Día 84, pero en ningún punto todos los perros quedaron libres de garrapatas adheridas. Las medias de los conteos en los perros tratados con lotilaner fueron significativamente menores que en los perros tratados con FSM en los Días 7, 42, 70 y 84 ($P < 0.05$). El porcentaje de eficacia en todas las visitas post-enrolamiento fue de 99.3 y 98.3% para los grupos de lotilaner y FSM, respectivamente ($t_{(190)} = 2.23$, $P = 0.0268$). Los dueños administraron exitosamente todos los tratamientos, y ambos productos fueron bien tolerados.

Conclusión: Bajo las condiciones de campo europeas, las tabletas masticables saborizadas de lotilaner se administraron mensualmente, y fueron > 98% efectivas al eliminar garrapatas vivas desde la primera evaluación post-tratamiento (Día 7) hasta el Día 56 y mantuvieron al 100% de los perros libres de garrapatas hasta los Días 70 y 84. Lotilaner fue seguro, proporcionando un control superior de garrapatas que FSM administrado de acuerdo con el mismo calendario.

Palabras clave: Credelio, *Dermacentor*, Perro, Eficacia, Europa, Campo, *Ixodes*, Lotilaner, *Rhipicephalus*, Garrapatas

Antecedentes

La amplia diseminación geográfica y la creciente abundancia en Europa de garrapatas ixodicas, *Ixodes ricinus*, *Dermacentor reticulatus* y *Rhipicephalus sanguineus* (*sensu lato*), se ha ligado a los cambios en el clima que han proporcionado condiciones epidemiológicas favorables, y a los cambios en los comportamientos humanos que aumentan el riesgo de exposición de ellos y sus mascotas [1-5]. Las garrapatas son una amenaza creciente para la salud humana y animal como resultado de un efecto patogénico directo y más importante aún, a través de los microorganismos causantes de enfermedad que ellas transmiten, incluyendo aquellos responsables de la encefalitis transmitida por garrapata, babesiosis, anaplasmosis y erliquiosis [5-7]. Para reducir el riesgo del establecimiento de infestaciones de garrapata canina, es, por lo tanto, importante que los veterinarios y los dueños tengan opciones

efectivas que puedan proporcionar de efectividad confiable y sostenida a través del intervalo entre tratamientos recomendado.

Una nueva opción para el control de garrapatas y pulgas es el compuesto de isoxazolina, lotilaner. Las isoxazolinas actúan sistémicamente para matar pulgas y garrapatas que infestan a los perros dirigiéndose a los distintos sitios de unión en el insecto y al ácido γ-aminobutírico (GABA)- y a los canales del ión cloro en las compuertas de glutamato acarinos para causar parálisis del parásito y la muerte [8, 9]. Se ha demostrado que lotilaner se une selectivamente a esos sitios en los invertebrados, pero no en los perros [9]. Otros miembros de la familia de parasiticidas de las isoxazolinas son el afoxolaner y el fluralaner que recibió primero una aprobación regulatoria para el uso en perros en el 2014, y sarolaner que fue aprobado para uso en perros en el 2015. Como el miembro más recientemente aprobado de la familia, lotilaner es un ectoparasiticida de rápido inicio que se presenta en una formulación de tabletas masticables saborizadas (Credelio™, Elanco) para perros. Los estudios de laboratorio demostraron que lotilaner comienza a matar rápidamente en las infestaciones inducidas con *I. ricinus*, y a sostener la actividad contra nuevas infestaciones de *Ixodes scapularis, I. ricinus, Dermacentor variabilis*, *D. reticulatus* y *R. sanguineus* (*s.l.*) por al menos 35 días, y *Amblyomma americanum* por al menos 28 días [10-12]. También se ha demostrado un inicio rápido similar y actividad sostenida contra las infestaciones por pulga [13, 14].

Para confirmar que estos resultados prometedores eran relevantes en las infestaciones naturales por garrapata, se diseñó un estudio para evaluar la seguridad y eficacia de lotilaner, tabletas masticables saborizadas, contra garrapatas en perros con dueño en Europa. En tres países, i.e. Alemania, Hungría y Portugal, se administró lotilaner oralmente por los dueños, una vez cada cuatro semanas por un total de tres tratamientos dentro del rango de dosis de 20.2 a 40.7 mg/kg de peso corporal en perros naturalmente infestados con garrapatas. Se utilizó una formulación tópica de fipronil/(S)-metopreno (FSM) (Frontline® Combo, Merial) como un control positivo para la comparación. Se evaluaron la seguridad y la eficacia de cada producto.

Métodos

Este estudio de campo con asesor-ciego, con control positive, aleatorizado, multicéntrico, clínico sin inferioridad se condujo en cumplimiento con los requisitos regulatorios nacionales y locales en Alemania, Hungría y Portugal; en cumplimiento con la guía VICH en Buenas Prácticas Clínicas (GCP; VICH GL 9) y la Directiva 2001/82/EC conforme a la enmienda; Las Normas que Gobiernan los

Productos Medicinales en la Unión Europea, Volumen VIIA: Guías para las pruebas de productos medicinales veterinarios: Demostración de Eficacia de Ectoparasiticidas, 7AE17a, página 215–222; EMEA/CVMP/EWP/ 005/2000-Rev.2: Guía para la prueba y evaluación de la eficacia de las sustancias antiparasitarias para el tratamiento y la prevención de la infestación de garrapatas y pulgas en perros y gatos, 12 Nov 2007; y consistente con las guías de la Asociación Mundial para el Avance de la Parasitología Veterinaria [15, 16].

Animales

Los perros se seleccionaron para el estudio en el Día 0 después de ser diagnosticados con infestaciones de al menos tres garrapatas vivas adheridas. Los conteos de garrapatas se llevaron a cabo en un solo perro en cada hogar (perro primario). Solamente los hogares con perros que satisficieran todos los criterios de inclusión y que no aplicara ninguno de los criterios de exclusión, se seleccionaron para ser incluidos. Los perros suplementarios en cualquiera de los hogares enrolados se incluyeron independientemente de la infestación de garrapatas. Para calificar para la inclusión, un hogar no podía tener más de tres perros, todos se requerían que estuvieran sanos, o con condiciones que se juzgaron que no interferirían con los objetivos del estudio, deberían de tener al menos ocho semanas de edad y pesar al menos 2 kg.

Se excluía un hogar del estudio si contenía perras gestantes o en lactancia, o que tuvieran intenciones de reproducción en los siguientes cuatro meses después de la última administración del tratamiento. Los hogares se eliminarían del estudio en cualquier momento a discreción del investigador o el patrocinador del estudio pro razones que incluían el no cumplimiento del protocolo (por ejemplo, tratamiento con algún producto de estudio-proscrito como cualquiera que tuviera eficacia contra garrapatas), la aparición de una enfermedad concomitante, o el desarrollo de un evento adverso severo que fuera incompatible con la continuación del estudio. Los perros suplementarios en cada hogar eran tratados con el mismo producto como los perros primarios.

Todos los perros se mantuvieron con sus dueños bajos sus condiciones usuales de alojamiento, durante y después del estudio. Debido a que FSM se aplicó tópicamente, se evitó el baño/ inmersión en agua al cabo de dos días después de la aplicación y baños más frecuentes que una vez a la semana. No se les permitió nadar a los perros en cauces de agua por dos días después de la aplicación, y se evitó que se les dieran baños más frecuentes que una vez por semana, cada que fuera posible y cada contacto con el agua tenía que ser documentado por el dueño.

Aleatorización y tratamiento

Dentro de cada clínica, los perros se aleatorizaron por hogar para los grupos de tratamiento en la secuencia de inclusión designada por el plan de aleatorización, usando un diseño de bloque y una proporción de 2:1 (lotilaner:FSM), con un total de enrolamiento objetivo de 180 perros primarios. El primer perro del hogar que presentara una infestación de al menos tres garrapatas era el perro primario en el que todos los conteos de garrapatas se usarían para los cálculos de eficacia. Todos los perros, incluyendo a los perros suplementarios del hogar se observaron para la evaluación de seguridad.

Todos los perros de cualquier hogar se aleatorizaron al mismo grupo de tratamiento:

- Los hogares del grupo 1 se les dispensó lotilaner en tabletas masticables saborizadas (Credelio, Elanco), disponibles en cinco tamaños de tabletas (56.25 mg, 112.5 mg, 225 mg, 450 mg y 900 mg de lotilaner), a ser administradas con base al peso corporal de cada perro del hogar para lograr un nivel de dosis entre un mínimo de 20.2 a un máximo de 40.7 mg/kg, en cumplimiento con la tabla de dosificación para el producto comercial. En la visita inicial y en la segunda y tercer visitas, el despachador de cada clínica proporcionó un número apropiado de tabletas para cada perro del hogar a ser tratado en una sola ocasión en cada uno de los Días 0, 28 (± 2), y 56 (± 2). Se les instruyó a los dueños que alimentaran a sus perros al cabo de 30 minutos antes del tratamiento;
- Los hogares del grupo 2 se les dispensó una formulación de fipronil 10%/(S)-metopreno 0.9% (Frontline Combo, Merial), disponible en cuatro tamaños (0.67 ml, 1.34 ml, 2.68 ml o 4.02 ml), para la aplicación en-casa en cada uno de los Días 0, 28 (± 2), y 56 (± 2). Se les instruyó a los dueños aplicar el producto de acuerdo a la etiqueta.

Evaluaciones del estudio

Los exámenes físicos y las mediciones de peso corporal se completaron en cada perro primario en cada visita, y en los perros suplementarios en los Días 0, y ± 2 días en cada uno de los Días 28, 56 y 84. Se colectaron muestras de sangre y orina de todos los perros para las evaluaciones de patología clínica en los Días 0 y 84 (o antes para los perros que salieron del estudio prematuramente).

Para los perros primarios, los conteos de garrapatas y la remoción por parte del personal clínico ciego, entrenados en los procedimientos del protocolo del estudio se completaron en el Día 7 (± 1), y ± 2 días en cada uno de los Días 14, 21, 28, 42, 56, 70 y 84. Las garrapatas adheridas vivas y muertas se colocaron en viales separados para su diferenciación posterior. La eficacia de cada producto fue evaluada con base a los conteos de garrapatas vivas adheridas en cada punto de tiempo. Para generar

una indicación de la presión ambiental de la garrapata durante el estudio, los números de perros y gatos diagnosticados como tratados para infestaciones por garrapatas en las clínicas de estudio fueron registrados.

Las tres poblaciones de estudio para las evaluaciones: la población de seguridad, que consistía de todos los perros, primarios y suplementarios, aleatorizados a un grupo de tratamiento y que recibieron al menos una dosis de cada producto de estudio; la población de intento-para-tratar (ITT) que consistía en todos los perros primarios en cada grupo de tratamiento; y la población per-protocolo (PP), que consistía de todos los perros primarios sin mayores violaciones al protocolo. El análisis de eficacia fue conducido tanto en las poblaciones ITT, como en las PP. Para sexo, edad, peso corporal, raza, crianza, el tiempo que pasa el animal dentro /fuera de casa, el campo, se calculó un resumen de estadísticas y/o frecuencias y los dos grupos se compararon con una prueba no-paramétrica (Kruskal-Wallis, Mann-Whitney, o la prueba exacta Fisher, dependiendo en el parámetro). La seguridad se evaluó de acuerdo a cualquier observación hecha por el dueño o el personal del estudio de eventos adversos referentes a cambios en el peso corporal, y a los resultados de laboratorio de urianálisis, hematología y química sérica.

La eficacia de cada tratamiento fue evaluada comparando la media base de los conteos de garrapatas en el Día 0 con aquellas de las visitas realizadas después de la administración del primer tratamiento y comparando la reducción general en la media de los conteos de garrapatas sobre todo el período de tratamiento. La eficacia se determinó con base al porcentaje de reducción en los conteos pre- a post- dosificación dentro de cada grupo de tratamiento. El porcentaje de eficacia en cada conteo en un punto de tiempo después de la dosificación se calculó como sigue:

Porcentaje de eficacia = 100 × (media del conteo de garrapatas Día 0 – media del conteo de garrapatas día real /media del conteo de garrapatas Día 0), en donde los conteos de garrapatas se basan en las garrapatas vivas adheridas.

Se llevaron a cabo los cálculos usando las medias geométricas y aritméticas. Los cálculos de las medias geométricas se realizaron tomando el logaritmo del conteo de garrapatas de cada perro. Sí alguno de los conteos era igual a cero, se agregó un “uno” al conteo por cada animal en el grupo y luego se substrajo de la media resultante previo al cálculo del porcentaje de eficacia. El análisis estadístico se completó para: el conteo de garrapatas para las garrapatas vivas adheridas; la reducción del conteo de garrapatas versus la base para las garrapatas vivas adheridas; las tasas de cura (porcentaje de perros con conteos de garrapatas de cero) para las garrapatas vivas adheridas.

Los grupos de tratamiento se compararon por medio de los métodos del análisis de (co)varianza (AN(C)OVA) sí la suposición de la distribución normal se satisfizo en la escala original o después de la posible transformación logarítmica. En el ANCOVA, el número de perros por hogar se usó como un covariado. La no-inferioridad se indicaba si el límite más bajo del intervalo de confianza de dos lados es del 95% (CI) para la proporción de los conteos de garrapatas para lotilaner, dividido por el mismo valor para FSM, proporcionó un 97.5% de confianza que los conteos de garrapatas del tratamiento de lotilaner no eran tan elevados como aquellos del tratamiento FSM, hasta el margen de no-inferioridad del 15%. La superioridad se indicó sí el 95% del CI cayó completamente dentro del intervalo (0, 1), proporcionando un 97.5% de confianza que los conteos de garrapatas del tratamiento de lotilaner fueron menores que aquellos del tratamiento de FSM. Sí la suposición de la distribución normal no se satisfizo se llevaron a cabo pruebas de Mann-Whitney U. Los números de AEs por signo clínico se compararon entre los grupos de tratamiento usando la prueba exacta de Fisher. Todos los cálculos se llevaron a cabo usando el software de SAS®, Versión 9.2.2.

Resultados

Perros y la eficacia contra las garrapatas

Ciento noventa y cinco perros primarios (hogares) se enrolaron en el estudio, 127 en el grupo de lotilaner y 68 en el grupo de FSM, en siete clínicas en Alemania, seis clínicas en Hungría, y seis clínicas en Portugal. El primer enrolamiento fue en Abril 14 de 2014, y el seguimiento final fue en Agosto 21 de 2014. Los dueños reportaron que administraron todos los tratamientos y fueron administrados exitosamente por los dueños. En toda la población de seguridad el nivel de dosis de lotilaner administrado tuvo un rango de 20.2 a 40.7 mg/kg. No hubo reportes de dueños que resultaron en la exclusión de un perro debido a la violación de un protocolo debido a la exposición al agua o al baño. Ninguno de los perros se excluyó completamente del análisis estadístico: tres perros primarios en cada grupo fueron excluidos parcialmente del análisis PP debido a un retraso en la visita del Día 84; once perros (seis primarios, cinco suplementarios) no completaron el estudio con respecto a los días en el calendario completo (día 84 ± 2). De los perros primarios que no completaron, un perro en el grupo de lotilaner y dos perros en el grupo FSM se retiraron debido a la falta de cumplimiento del dueño con el protocolo; y dos perros en el grupo de lotilaner y uno en el grupo de FSM se retiraron debido a severos eventos adversos (AEs), detallado en la sección de Seguridad.

Los grupos son homogéneos para la distribución de edad, peso y sexo, y no hubo diferencias base estadísticamente significativas entre los grupos de tratamiento en cualquier variable demográfica (Tabla 1). Cuarenta y ocho razas diferentes se incluyeron en el estudio, de los cuáles la raza más frecuentemente enrolada fue el Labrador retriever (*n* = 12), Pastor Alemán (*n* = 9), y Golden retriever (*n* = 8). Las especies de garrapatas infestando perros en cada grupo se balancearon, sin diferencia significativa entre grupos. A lo largo del estudio, en cada país el número de perros, no estudiados, que fueron tratados para las infestaciones de garrapatas en las clínicas participantes verificaron la presencia de un desafío de garrapatas a lo largo del período de estudio (Fig. 1). Se realizaron observaciones similares en las clínicas del estudio de tratamientos de gatos para las infestaciones de garrapatas en gatos en cada país y a lo largo del período de estudio.

En los perros del estudio, las especies de garrapatas más comúnmente identificadas fueron *I. ricinus*, *R. sanguineus* (*s.l.*) y *D. reticulatus*, con *Ixodes hexagonus* también encontrada en 12 perros del estudio (Tabla 2). En Alemania, la especie más comúnmente aislada fue *I. ricinus*, con identificación ocasional de *I. hexagonus* y *D. reticulatus.* Hubo una identificación de solo una *R. sanguineus* (*s.l.*) de un perro en Alemania. En Hungría, tanto *I. ricinus* y *D reticulatus* fueron comúnmente identificadas, se colectaron garrapatas *R. sanguineus* (*s.l.*) de tres perros y hubo una sola identificación de *Haemaphysalis concinna*. En Portugal, en donde los conteos de garrapatas en cada perro fueron mucho mayores que en otros países, la especie de garrapata dominante fue *R. sanguineus* (*s.l.*). En este país, se encontraron otras especies en solo dos perros, una un macho y una hembra de *D. reticulatus*, y una con 20 hembras de *I. hexagonus*. A pesar del tratamiento con FSM, este perro permaneció infestado con *I. hexagonus* en diferentes puntos hasta el Día 84, cuando se recuperaron dos ninfas vivas. Los diez perros del estudio tratados con lotilaner presentados en el Día 0 con al menos un *I. hexagonus* (todos en Alemania) estaban libres de esta especie en todas las evaluaciones subsecuentes.

No hubo diferencia estadísticamente significativa entre la población de ITT y PP, y solamente los resultados de eficacia en la población PP se presentaron aquí. La media geométrica base de los conteos de garrapatas en los grupos de lotilaner y fipronil fueron de 6.06 y 5.01, respectivamente (Tabla 3). En la primera evaluación post-tratamiento (Día 7) la media geométrica del conteo de garrapatas para los perros tratados con lotilaner (0.07) fue significativamente menor ($t_{(190)} = 2.37$, $P = 0.0190$) que los perros tratados con FSM (0.17), y permanecieron más bajos en todos menos en uno de los conteos hasta la evaluación final en el Día 84. Otros puntos en los que la media de los conteos en el grupo de lotilaner fue significativamente más bajo que los conteos del grupo de FSM fueron los Días 42 ($t_{(186)} = 2.46$, $P = 0.0148$), 70 ($t_{(184)} = 2.04$, $P = 0.0425$), y 84 ($t_{(178)} = 2.33$, $P = 0.0209$). En el grupo

de lotilaner, la eficacia basada en las medias geométricas permaneció mayor a un 98% (basado en las medias aritméticas al menos un 95%) a lo largo del estudio, mientras que en el grupo de FSM la eficacia permaneció cuando más a 96% (para medias aritméticas mayores que 93%) (Tabla 4). Más de un 95% de los perros tratados con lotilaner estaban libres de garrapatas vivas adheridas a partir de la primera evaluación (Día 7) al Día 63, y en los Días 70 y 84 todos los perros en este grupo estaban completamente libres de garrapatas vivas (Fig.2). La investigación de la eficacia contra las diferentes especies infestantes de garrapatas en los perros del estudio no revelaron ninguna desviación clínica relevante de los resultados generales de la eficacia contra garrapatas de ambos productos.

En el grupo de lotilaner, el porcentaje promedio en la reducción en los conteos de las garrapatas vivas adheridas en todas las visitas post-enrolamiento fue del 99.3% (media geométrica) y 98.6% (media aritmética), y para el grupo FSM 98.3 y 97.4%. Se demostró que (con un 97.5% de confianza límite) que los conteos de garrapatas en el grupo de lotilaner no fueron más elevados que los conteos de garrapatas en el grupo FSM, hasta un margen de no-inferioridad de 15%. Además de demostrar la no-inferioridad, estos resultados también mostraron superioridad de lotilaner sobre FSM ($t_{(190)}$ = 2.23, P = 0.0268).

Dos perros que fueron asignados aleatoriamente al grupo de lotilaner tuvieron cargas pre-tratamiento de más de 200 *R. sanguineus* (*s.l.*). Uno de estos perros estaba libre de garrapatas vivas adheridas en la primer visita post-tratamiento (Día 7) y permaneció así, de ahí en adelante, y el otro permaneció libre de garrapatas vivas adheridas siguiendo la administración del segundo tratamiento. En el grupo FSM, el conteo base de garrapatas más elevado fue de 64 (*R. sanguineus* (*s.l.*), y en este perro hubieron hallazgos ocasionales de números bajos de esta especie a lo largo del estudio.

Seguridad

Incluyendo a los perros suplementarios en cada hogar, la población de seguridad comprendió a 192 perros tratados con lotilaner y 94 con FSM. Ambos tratamientos de estudio fueron bien tolerados. Los AEs más comúnmente reportados fueron desórdenes del tracto digestivo (diarrea, emesis, dilatación gástrica) que ocurrió en 3.6% y 1.1% en el grupo de lotilaner- y FSM-, respectivamente (Tabla 5). No hubo diferencias estadísticas entre-grupo en ninguno de estos signos. Un perro del grupo de lotilaner, una hembra Dachshund pelo largo de 14 años de edad, tenia una historia en el enrolamiento de una sospecha de nefritis (crónica) basándose en la prueba del Día 0 (azotemia). En el Día 20, el perro presentó polidipsia y poliuria pero no recibió tratamiento concomitante excepto un cambio a una dieta renal especializada. A partir del Día 28 no se observaron polidipsia/poliuria, pero un análisis de sangre

adicional reveló una incremento en la creatinina sérica. Esto se registró como otro AE (resultado de prueba anormal – azotemia) en el Día 30 debido a que empeoraba en comparación con el Día 0. Al final del estudio, la azotemia no empeoró significativamente, ni tampoco se resolvió, mientras que los niveles de fosfato y potasio aumentaron significativamente, llevando a la documentación de dos AEs más. No hubieron diferencias estadísticas significativas entre los dos grupos de tratamiento en ninguno de los signos clínicos reportados de AEs o AEs severos.

Se presentaron cuatro AEs severos observados en los perros del estudio, ninguno de los cuáles fue atribuido al tratamiento. En el grupo de lotilaner, dos perros primarios se afectaron, uno de los cuáles presentaba el síndrome de cauda equina en el Día 5 y se le practicó eutanasia ante la petición del dueño; el otro murió después de estar involucrado en un accidente de automóvil. Un perro del grupo de lotilaner suplementario con una cardiomiopatía dilatativa pre-estudio fue encontrado muerto por el dueño en el Día 75. En el grupo de FSM, un perro primario permaneció en el estudio después de recuperarse de secuelas de una cirugía para corregir una paresis del miembro anterior debido a un prolapso iniciando en el Día 27.

No se observó una diferencia significativa entre los grupos de tratamiento para los resultados de hematología o urianálisis. Los resultados de la química en suero mostraron una diferencia significativa para el colesterol en el día 84, con valores menores en el grupo de FSM ($t_{(163)} = 2.81$, $P = 0.0056$), pero la media del valor permaneció dentro del rango normal de referencia y no se asoció con ningún signo clínico de enfermedad. Todos los otros parámetros medidos no fueron diferentes significativamente.

La única diferencia significativa en peso corporal entre los dos grupos de tratamiento fue en el Día 7 ($t_{(189)} = 2.19$, $P = 0.0301$) cuando el grupo de lotilaner tuvo una media del peso más alta que el grupo de FSM (corregido para el peso corporal base). La media del peso corporal para cada grupo de tratamiento no cambió notablemente durante el período de estudio pero aumentó ligeramente del día 0 al día 84 en ambos grupos.

Las vacunaciones multivalentes y monovalentes contra la rabia y las medicaciones concomitantes se administraron a los perros del grupo de lotilaner sin eventos adversos asociados. Las medicaciones sistémicas concomitantes reportadas fueron amoxicilina, penicilina benzilprocaínica / dihidroestreptomicina, clorhidrato de benazepril, domperidona, furosemida, imepitoin, meloxicam, milbemicina oxima, pimobendan, praziquantel, praziquantel/pirantel pamoata/febantel, robenacoxib, y espironolactona.

Discusión

La frecuencia de las infestaciones de garrapatas en este estudio sustenta el riesgo continuo del desafío de garrapatas a lo largo de un rango de climas en Europa, y enfatiza la necesidad para un tratamiento efectivo contra la garrapata y medidas preventivas. Hubo un desafío sostenido de garrapatas para enrolar a los perros apoyado por la presentación de perros infestados con garrapatas que no pertenecían al estudio en las clínicas participantes a lo largo del estudio, y a la persistencia de los números de garrapatas en los perros tratados. Adicionalmente, la mayoría de los perros se clasificaron como perros de exterior de ambientes rurales, situaciones en donde los perros son más probablemente expuestos al desafío de garrapatas.

Las especies dominantes que fueron identificadas en este estudio, i.e. *I. ricinus, R. sanguineus* (*s.l.*) y *D. reticulatus*, son consistentes con otros reportes, aunque no hay soporte para la sugerencia de una migración hacia el norte de *R. sanguineus* (*s.l.*) más allá de áreas mediterráneas [17-19]. Hubo diferencias regionales definidas en el desafío, con *Ixodes*, predominantemente *I. ricinus* pero también *I. hexagonus,* la especie más comúnmente observada en Alemania, un balance entre *I. ricinus* y *D. reticulatus* en Hungría, y *R. sanguineus* fue dominante en Portugal. Los conteos totales de garrapatas en los perros de Portugal debidos a infestaciones por *R. sanguineus* (*s.l.*) fueron mucho mayores en número que aquellos que se presentaron con otras especies en Alemania y en Hungría.

El hecho de que todos los perros fueron tratados exitosamente por sus dueños, sin preocupaciones por falta de cumplimiento indica que lotilaner en su formulación de tabletas masticables saborizadas es fácilmente aceptada por los perros. Esta conclusión es apoyada por otro estudio de campo europeo en donde se determinó que lotilaner es palatable para los perros, y un estudio de campo en Estados Unidos en donde no hubo diferencia práctica entre la aceptación voluntaria de lotilaner y afoxalaner en tratamientos ofrecidos por los dueños [20, 21].

Independientemente del país, región o especie de garrapata infestante o del tamaño de la carga de garrapatas, lotilaner proporcionó una alta y sostenida eficacia a lo largo del estudio de 84 días. Al final del estudio todos los perros tratados con lotilaner estaban completamente libres de garrapatas vivas, y la media geométrica de los conteos de garrapatas eran significativamente más bajos que aquellos del grupo FSM en general y en los Días 7, 42 y 70. La elevada eficacia sostenida contra las infestaciones naturales de garrapatas confirma los estudios con infestaciones inducidas en los que la eficacia contra las tres especies dominantes de garrapatas en Europa se había demostrado que era de al menos un 95% a lo largo de 35 días después del tratamiento [11]. Lotilaner fue bien tolerado entre todas las razas y ambientes, y fue administrado con seguridad en un rango de medicaciones comunes y

vacunas. Estos resultados son consistentes con un estudio de campo paralelo en Europa evaluando la eficacia de lotilaner contra las pulgas [20]. En ese estudio un 97.5% de los perros tratados con lotilaner eliminó a las pulgas vivas al cabo de cuatro semanas después del tercer mes de un tratamiento trimestral, y lotilaner fue significativamente más efectivo que fipronil.

En este estudio, la media aritmética de las reducciones del conteo de garrapatas en el grupo FSM tuvo un rango de 93.7% en el Día 7 a un 99.5% en el Día 84. Estos resultados son similares a aquellos reportados en dos estudios de campo similares en Europa comparando fipronil a otras isoxazolinas. En una comparación con sarolaner, la media aritmética del porcentaje de reducciones en el grupo de fipronil tuvo un rango de 88.5 a 98.1%, con sarolaner mostrando reducciones significativamente mayores en los conteos de garrapatas en dos ocasiones (Días 30 y 60) [17]. En un estudio comparativo con fluralaner, la eficacia anti-garrapata de fipronil basada en las medias geométricas tuvo un rango del 97.6 al 100%, sin diferencias significativas entre los grupos [18]. Por lo tanto, los resultados acumulativos de estos estudios sugieren que mientras la actividad para matar rápido a la garrapata de fipronil permanece alta, las isoxazolinas tienen el potencial de proveer un nivel superior del control, que puede ser de relevancia en la prevención de la transmisión de patógenos transmitidos por las garrapatas y en la percepción de la efectividad del producto por el dueño.

Conclusión

Los resultados de este estudio, llevado a cabo en un rango diverso de perros con dueño y regiones geográficas, se demostró que bajo un amplio rango de condiciones de campo en Europa, un solo tratamiento con lotilaner resulta en una reducción del 98.9% en los conteos de garrapatas vivas adheridas para el momento de la primera evaluación a los siete días después del primer tratamiento. Este alto nivel de eficacia fue sostenido después de tres tratamientos mensuales. Al final del estudio, 28 días después del tratamiento final, todos los perros tratados con lotilaner estaban libres de garrapatas vivas. Los resultados demuestran que lotilaner tuvo una alta eficacia consistente y sostenida contra las garrapatas bajo las condiciones europeas. Los resultados también demostraron que lotilaner es fácil de administrar por los dueños, y es bien tolerado y seguro para usarse con un rango de vacunaciones y medicaciones concomitantes. El control de la garrapata proporcionado por lotilaner fue superior al proporcionado por una formulación aplicada tópicamente de fipronil/(S)-metopreno.

Abreviaturas

AE: evento adverso; AN(C)OVA: análisis de (co)varianza; CI: intervalo de confianza; EMEA/CVMP: EMEA/CVMP/EWP Agencia Europea de Medicinas/ Comité para Productos Medicinales de Uso Veterinario; GABA: ácido γ-aminobutírico; ITT: intento-para-tratar; PP: per-protocolo; DE: desviación estándar; VICH: Cooperación Internacional para la Armonización de los Requisitos Técnicos para el Registro de los Productos Medicinales Veterinarios

Aprobación de ética y consentimiento para participar

El estudio fue aprobado por el Grupo de Bienestar Animal de Novartis Salud Animal Global. A los dueños de perros participantes se les solicitó que firmaran una forma de consentimiento con información para que su perro(s) participaran en el estudio, después de los detalles del estudio, el diseño y productos bajo investigación se explicaron.

Consentimiento para la publicación

No aplicable.

Disponibilidad de datos y material

Los grupos de datos apoyan las conclusiones de este artículo están incluidos en el artículo. Debido a la confidencialidad comercial de la investigación , los datos no incluidos en el manuscrito solo pueden estar disponibles a investigadores *bona fide* sujetos a un acuerdo de no-divulgación.

Fondos

Este estudio fue patrocinado por Elanco.

Intereses competentes

DC, WS, MM, JD y SN son empleados de Elanco Animal Health.

Contribuciones de los autores

Todos los autores participaron en el diseño y finalización de los estudios y se involucraron en el manuscrito preliminar. Todos los autores leyeron y aprobaron el manuscrito final.

Reconocimientos

Los autores quieren agradecer al Dr. Bill Ryan de Ryan Mitchell Associates LLC por su apoyo con el manuscrito.

Detalles del autor

[1]Elanco Animal Health, Schwarzwaldallee 215, CH-4058 Basilea, Suiza, WRO-1032.2.58. [2]Elanco Animal Health, 2500 Innovation Way, Greenfield, IN 46140

Referencias

1. Jaenson TG, Jaenson DG, Eisen L, Petersson E, Lindgren E. Changes in the geographical distribution and abundance of the tick *Ixodes ricinus* during the past 30 years in Sweden. Parasit Vectors. 2012;5:8.
2. Zając Z, Katarzyna B, Buczek A. Factors influencing the distribution and activity of *Dermacentor reticulatus* (F.) ticks in an anthropopressure-unaffected area in central-eastern Poland. Ann Agric Environ Med. 2016;23:270-5.
3. Abdullah S, Helps C, Tasker S, Newbury H, Wall R. Ticks infesting domestic dogs in the UK: a large-scale surveillance programme. Parasit Vectors. 2016;9:391.
4. Földvári G, Široký P, Szekeres S, Majoros G, Sprong H. *Dermacentor reticulatus*: a vector on the rise. Parasit Vectors. 2016;9:314.
5. Randolph SE. Evidence that climate change has caused 'emergence' of tick-borne diseases in Europe? Int J Med Microbiol. 2004;293 Suppl 37:5-15.
6. Kilpatrick AM, Randolph SE. Drivers, dynamics, and control of emerging vector-borne zoonotic diseases. Lancet. 2012;380:1946-55.
7. Jaenson TG, Hjertqvist M, Bergström T, Lundkvist A. Why is tick-borne encephalitis increasing? A review of the key factors causing the increasing incidence of human TBE in Sweden. Parasit Vectors. 2012;5:184.
8. Ozoe Y, Asahi M, Ozoe, F, Nakahira K, Mita T. The antiparasitic isoxazoline A1443 is a potent blocker of insect ligand-gated chloride channels. Biochem Biophys Res Commun. 2010;391;744-9.
9. Rufener L, Danelli V, Bertrand D, Sager H. The novel isoxazoline ectoparasiticide lotilaner (Credelio™): a non-competitive antagonist specific to invertebrates γ-aminobutyric acid-gated chloride channels (GABACls). Parasit Vectors (In press).
10. Murphy M, Cavalleri D, Seewald W, Drake J, Nanchen S. Laboratory evaluation of the speed of kill of lotilaner (Credelio™) against *Ixodes ricinus* ticks on dogs. Parasit Vectors. 2017 (In press).

11. Cavalleri D, Murphy M, Gorbea RL, Seewald W, Drake J, Nanchen S. Laboratory evaluations of the immediate and sustained efficacy of lotilaner (Credelio^{TM}) against three common species of ticks affecting dogs in Europe. Parasit Vectors. 2017 (In press).
12. Murphy M, Garcia R, Karadzovska D, Cavalleri D, Snyder D, Seewald W, et al. Laboratory evaluations of the immediate and sustained effectiveness of lotilaner (Credelio^{TM}) against four common species of ticks affecting dogs in North America. Parasit Vectors. 2017 (In press).
13. Cavalleri D, Murphy M, Seewald W, Drake J, Nanchen S. Assessment of the onset of lotilaner (Credelio^{TM}) speed of kill of fleas on dogs. Parasit Vectors. 2017 (In press).
14. Cavalleri D, Murphy M, Seewald W, Drake J, Nanchen S. Assessment of the speed of flea kill of lotilaner (Credelio^{TM}) throughout the month following oral administration to dogs. Parasit Vectors. 2017 (In press).
15. European Medicines Agency, Committee for Medicinal Products for Veterinary Use, 2000. Guideline on Good Clinical Practices. VICH Topic GL9 http://www.ema.europa.eu/docs/en GB/document library/Scientific guideline/2009/10/WC500004343.pdf. Accessed January 12, 2017.
16. Marchiondo AA, Holdsworth PA, Fourie LJ, Rugg D, Hellmann K, Snyder DE, et al. World Association for the Advancement of Veterinary Parasitology (W.A.A.V.P.) 2nd. ed.: Guidelines for evaluating the efficacy of parasiticides for the treatment, prevention and control of flea and tick infestations on dogs and cats. Vet Parasitol. 2013;194:84–97.
17. Becskei C, De Bock F, Illambas J, Mahabir SP, Farkas R, Six RH. Efficacy and safety of a novel oral isoxazoline, sarolaner (Simparica™) in the treatment of naturally occurring flea and tick infestations in dogs presented as veterinary patients in Europe. Vet Parasitol. 2016;222:49-55.
18. Rohdich N, Roepke RK, Zschiesche E. A randomized, blinded, controlled and multi-centered field study comparing the efficacy and safety of Bravecto (fluralaner) against Frontline (fipronil) in flea- and tick-infested dogs. Parasit Vectors. 2014;7:83.
19. Beugnet F, Marié JL. Emerging arthropod-borne diseases of companion animals in Europe. Vet Parasitol. 2009;163:298-305.
20. Cavalleri D, Murphy M, Seewald W, Drake J, Nanchen S. A randomized, controlled field study to assess the efficacy and safety of lotilaner tablets (Credelio^{TM}) in controlling fleas in client-owned dogs in Europe. Parasit Vectors. 2017 (In press).
21. Karadzovska D, Chappell K, Coble C, Murphy M, Cavalleri D, Wiseman S, Drake J, Nanchen S. A randomized, controlled field study to assess the efficacy and safety of lotilaner flavored chewable

tablets (Credelio™) in eliminating fleas in client-owned dogs in the USA. Parasit Vectors, 2017 (In press).

Leyendas de las figuras

Fig. 1 Media del número de perros que no estaban en el estudio tratados para infestaciones por garrapata; promedio de los sitios de estudio dentro de los países en intervalos semanales a lo largo del estudio

Fig. 2 Porcentaje de perros que estaban libres de garrapatas vivas después del tratamiento con lotilaner o fipronil/(S)-metopreno en los Días 0, 30 y 60

Tabla 1 Demografía de los perros enrolados (población de eficacia)

		**Lotilaner (*n* = 127)**	**Fipronil/(S)-metopreno (*n* = 68)**
Edad (años)	Media ± DE	4.6 ± 3.5	5.1 ± 4.0
	Rango	0.2–14.0	0.3–16.0
Peso (kg)	Media ± DE	22.1 ± 13.8	20.4 ± 13.6
	Rango	2.4–62.0	2.2–73.9
Sexo	Hembra	47 (37.0%)	27 (39.7%)
	Macho	80 (63.0%)	41 (60.3%)
Lugar	Campo	83 (65.4%)	48 (70.6%)
	Urbano	44 (34.6%)	20 (29.4%)
Perro primario pasa tiempo:			
	Principalmente interior	41 (32.3%)	26 (38.2%)
	Principalmente exterior	86 (67.7%)	42 (61.8%)

Abreviatura: DE, desviación estándar

Tabla 2 Porcentaje de perros del estudio infestados con diferentes especies de garrapatas en la base

	**Lotilaner (*n* = 127)**		**Fipronil/(S)-metopreno (*n* = 68)**	
	**% infestado**	**Total de garrapatas vivas**	**% infestado**	**Total de garrapatas vivas**
Ixodes ricinus	64.6	294	60.3	136
Rhipicephalus sanguineus	36.2	1147	33.8	216
Dermacentor reticulatus	27.6	85	32.4	58
Ixodes hexagonus	7.9	14	2.9	23
Ixodes spp.	0.8	1	1.4	1
Haemaphysalis concinna	0.8	1	0	0

Tabla 3 Media de los conteos de garrapatas vivas adheridas en cada grupo de tratamiento (población per protocolo)

	**Día del estudio**								
	**0**	**7**	**14**	**21**	**28**	**42**	**56**	**70**	**84**
Lotilaner (*n* = 127)									
Media geométrica	6.06	0.07[a]	0.07	0.10	0.06	0.02[b]	0.05	0.00[c]	0.00[d]
Media aritmética (DE)	11.61 (28.76)	0.15 (0.94)	0.15 (0.76)	0.58 (5.27)	0.15 (0.82)	0.04 (0.30	0.28 (2.78)	0.00 (0.00)	0.00 (0.00)
Fipronil/(S)-metopreno (*n* = 68)									
Media geométrica	5.01	0.17[a]	0.11	0.13	0.09	0.11[b]	0.04	0.02[c]	0.03[d]
Media aritmética (DE)	6.31 (8.06)	0.40 (1.47)	0.19 (0.63)	0.22 (0.65)	0.18 (0.89)	0.20 (0.68)	0.06 (0.30)	0.03 (0.17)	0.05 (0.28)

Números dentro de columnas con el mismo superíndice son diferentes significativamente: $^{a}t_{(190)} = 2.37$, $P = 0.0190$; $^{b}t_{(186)} = 2.46$, $P = 0.0148$; $^{c}t_{(184)} = 2.04$, $P = 0.0425$; $^{d}t_{(178)} = 2.33$, $P = 0.0209$

Abreviatura: DE, desviación estándar

Tabla 4 Porcentaje de efectividad de lotilaner y fipronil/(S)-metopreno (población per protocolo)

	**Día de estudio**							
	**7**	**14**	**21**	**28**	**42**	**56**	**70**	**84**
Lotilaner (*n* = 127)								
Media geométrica	98.9[a]	98.9	98.4	98.9	99.6[b]	99.2	100.0	100.0
Media aritmética	98.7	98.7	95.0	98.7	99.7	97.6	100.0	100.0
Fipronil/(S)-metopreno (*n* = 68)								
Media geométrica	96.6[a]	97.8	97.3	98.3	97.9[b]	99.2	99.6	99.4
Media aritmética	93.7	96.9	96.5	97.2	96.9	99.0	99.5	99.2

Números dentro de las columnas con el mismo superíndice son diferentes significativamente:[a]$t_{(190)} = 3.14$, $P = 0.0020$; [b]$t_{(186)} = 2.39$, $P = 0.0180$

Tabla 5 Eventos adversos observados en al menos 1% de los perros en cada grupo

	**Lotilaner (*n* =192)**	**Fipronil/(S)-metopreno (*n* = 94)**	**Comparación (Prueba exacta de Fischer)**
Emesis	3.1%	0.0%	z = 1.73, P = 0.1825
Diarrea	1.0%	0.0%	z = 0.99, P = 0.5572
Dilatación gástrica	0.0%	1.1%	z = 1.42, P = 0.3287
Resultado anormal de prueba	1.6%	2.1%	z = 0.34, P = 1.0000
