# Supplementary material for: A randomized, controlled study to assess the efficacy and safety of lotilaner (Credelio™) in controlling ticks in client-owned dogs in Europe
Source: Parasit Vectors. 2017 Nov 1;10:531. doi: 10.1186/s13071-017-2478-9 (PMC5664821; doi:10.1186/s13071-017-2478-9)
Supplement: Supplementary file 2 — French translation of the Abstract. (PDF 37 kb) [file 13071_2017_2478_MOESM2_ESM.pdf]

Étude contrôlée randomisée visant à évaluer l'efficacité et l'innocuité du lotilaner (Credelio™) dans le contrôle des tiques chez des chiens de compagnie en Europe

Daniela Cavalleri[1], Martin Murphy[1], Wolfgang Seewald[1], Jason Drake[2]* et Steve Nanchen[1]

[1]Elanco Santé animale, Mattenstrasse 24a, CH-4058 Bâle, WRO-1032.2.58, Suisse

[2]Elanco Santé animale, 2500 Innovation Way, Greenfield, IN 46140, États-Unis

*Correspondance : drake_jon_j@elanco.com

Adresse électronique :

Daniela Cavalleri[1]	Adresse électronique : cavalleri_daniela_a@elanco.com
Martin Murphy[1]	Adresse électronique : murphy_martin_gerard@elanco.com
Wolfgang Seewald[1]	Adresse électronique : seewald_wolfgang@elanco.com
Jason Drake[2]	Adresse électronique : drake_jon_j@elanco.com
Steve Nanchen[1]	Adresse électronique : nanchen_steve@elanco.com

Résumé

Contexte : il a été démontré que l'administration orale de comprimés à croquer aromatisés à base de lotilaner (Credelio™, Elanco) exerce un effet létal rapide sur les tiques infestant le chien, avec une efficacité persistant au moins 35 jours. Une étude a été conduite en Europe afin de confirmer l'innocuité et l'efficacité contre les tiques du lotilaner chez des chiens de compagnie.

Méthodes : les chiens de cette étude conduite en aveugle, ont été recrutés dans 19 cliniques en Allemagne, en Hongrie et au Portugal. Les foyers sélectionnés, ne comptant pas plus de trois chiens, ont été randomisés selon un rapport proche de 2/1 dans un des deux groupes de traitement suivants : lotilaner ou fipronil/(S)-méthoprène (FSM) (Frontline® Combo Spot-on, Merial). Dans chaque foyer, un chien hébergeant au moins trois tiques fixées et vivantes, a été sélectionné comme chien principal. Les traitements ont été délivrés aux propriétaires qui devaient les administrer à J0, J28 (± 2) et J56 (± 2) à tous les chiens du foyers. Un comptage des tiques a été réalisé sur les chiens principaux à J7 (± 1), et à J14, J21, J28, J42, J56, J70 et J84 ± 2 jours ; la sécurité a été évaluée chez les autres chiens à

J28, J56 et J84 ± 2 jours. L'efficacité était évaluée en comparant le nombre de tiques fixées et vivantes à J0 à celui des comptages suivants.

Résultats : les espèces de tiques les plus fréquemment extraites étaient *Ixodes ricinus, Dermacentor reticulatus* et *Rhipicephalus sanguineus (sensu lato),* l'espèce *Ixodes hexagonus* ayant également été isolée. Dans le groupe lotilaner (n = 127) la diminution du nombre de tiques (moyenne géométrique) était d'au moins 98 % entre la première visite suivant le traitement (J7) et J56, où l'efficacité était de 100 %. L'efficacité du FSM (n = 68) est restée d'au moins 96 % jusqu'à J84, mais il n'a jamais été observé d'absence totale de tiques fixées et vivantes chez l'ensemble des chiens. Le nombre moyen de tiques était significativement inférieur chez les chiens traités par lotilaner par rapport au groupe sous FSM à J7, J42, J70 et J84 ($p < 0{,}05$). L'efficacité (en pourcentage) mesurée pour toutes les visites suivant l'inclusion était respectivement de 99,3 % et 98,3 % dans les groupes lotilaner et FSM ($t_{(190)} = 2{,}23$, p = 0,0268). Les propriétaires ont réussi à administrer tous les traitements et les deux produits ont été bien tolérés.

Conclusion : en Europe, dans des conditions réelles d'infestation, l'administration mensuelle de comprimés à croquer aromatisés à base de lotilaner a permis d'éliminer les tiques vivantes avec une efficacité > 98 % entre la première évaluation réalisée après traitement (J7) et J56, 100 % des chiens ne présentant aucune tique à J70 et J84. Le traitement par le lotilaner a été bien toléré et a permis d'obtenir un meilleur contrôle des tiques par rapport au FSM, administré selon le même schéma.
